# Recovering individual haplotypes and a contiguous genome assembly from pooled long-read sequencing of the diamondback moth (Lepidoptera: Plutellidae)

**DOI:** 10.1093/g3journal/jkac210

**Published:** 2022-08-18

**Authors:** Ivy Whiteford, Arjen E van’t Hof, Ritesh Krishna, Thea Marubbi, Stephanie Widdison, Ilik J Saccheri, Marcus Guest, Neil I Morrison, Alistair C Darby

**Affiliations:** Institute of Integrative Biology, University of Liverpool, Liverpool L69 7ZB, UK; Institute of Integrative Biology, University of Liverpool, Liverpool L69 7ZB, UK; Institute of Integrative Biology, University of Liverpool, Liverpool L69 7ZB, UK; IBM Research UK, STFC Daresbury Laboratory, Warrington WA4 4AD, UK; Oxitec Ltd., Abingdon OX14 4RQ, UK; General Bioinformatics, Jealott's Hill International Research Centre, Bracknell RG42 6EY, UK; Institute of Integrative Biology, University of Liverpool, Liverpool L69 7ZB, UK; Syngenta, Jealott's Hill International Research Centre, Bracknell, RG42 6EY, UK; Oxitec Ltd., Abingdon OX14 4RQ, UK; Institute of Integrative Biology, University of Liverpool, Liverpool L69 7ZB, UK

**Keywords:** pool-seq, haplotype, assembly, *Plutella xylostella*

## Abstract

The assembly of divergent haplotypes using noisy long-read data presents a challenge to the reconstruction of haploid genome assemblies, due to overlapping distributions of technical sequencing error, intralocus genetic variation, and interlocus similarity within these data. Here, we present a comparative analysis of assembly algorithms representing overlap-layout-consensus, repeat graph, and de Bruijn graph methods. We examine how postprocessing strategies attempting to reduce redundant heterozygosity interact with the choice of initial assembly algorithm and ultimately produce a series of chromosome-level assemblies for an agricultural pest, the diamondback moth, *Plutella xylostella* (L.). We compare evaluation methods and show that BUSCO analyses may overestimate haplotig removal processing in long-read draft genomes, in comparison to a k-mer method. We discuss the trade-offs inherent in assembly algorithm and curation choices and suggest that “best practice” is research question dependent. We demonstrate a link between allelic divergence and allele-derived contig redundancy in final genome assemblies and document the patterns of coding and noncoding diversity between redundant sequences. We also document a link between an excess of nonsynonymous polymorphism and haplotigs that are unresolved by assembly or postassembly algorithms. Finally, we discuss how this phenomenon may have relevance for the usage of noisy long-read genome assemblies in comparative genomics.

## Introduction

Technical and analytical advances in genomics have dramatically improved the achievable standard of genome projects. The amount of high molecular weight (HMW) DNA required to perform long-read sequencing has reduced significantly and has been accompanied by a steady increase in sequencing read lengths and read accuracy (Kingan *et al.* 2019). Longer reads have aided genome assembly efforts by providing information linking unique genomic sequences flanking repetitive elements, which represented a challenge to algorithms reliant on short-read data (Koren *et al.* 2017). However, early long-read methods, such as Oxford nanopore and SMRT sequencing contained higher error rates in raw sequence reads (Derrington *et al.* 2010; Chin *et al.* 2013). Whilst this technical noise can be tolerated by various means (Chin *et al.* 2013, 2016; Koren *et al.* 2017; Kolmogorov *et al.* 2019; Ruan and Li 2020), it is ultimately confounded with the real biological variation present in the underlying samples. The form this biological variation takes and the way it is distributed across the genome of an organism can influence the accuracy of a reconstructed haploid (or phased diploid) genome assembly (Kajitani *et al.* 2019). Various assembly pipelines and algorithms have been explicitly designed to overcome challenges of heterozygosity (Chin *et al.* 2016; Huang *et al.* 2017; Roach *et al.* 2018), repeat resolution (Kolmogorov *et al.* 2019), speed (Ruan and Li 2020), and integration of multiple data types (Ye *et al.* 2016; Zimin *et al.* 2017). Furthermore, all assembly software have some level of parameterization available to optimize results, yielding a huge array of possible outcomes.

Alongside these computational innovations, several experimental approaches and supplemental data types can augment existing data. Trio-sequencing can partition heterozygous variation in an F1 individual using information from parental haplotypes (Koren *et al.* 2018). Linked-reads utilize microfluidics to uniquely barcode reads that derive from discrete large DNA fragments, thereby capturing longer-range information than standard short-read preparations do not (Zheng *et al.* 2016). Chromosome conformation capture (Hi-C) data crosslinks in vivo chromatin molecules and recovers pairs of reads that derive from these crosslinks, producing data that reflects the 3D organization of the nucleus, and also long-range cis-chromosome associations (Ghurye *et al.* 2019). In addition to the proliferation of supporting data types, the efficacy and quality of core genomic data have improved. Improvements to data quality predominantly come from platform advancements such as high-fidelity (HiFi) long reads (Nurk *et al.* 2020; Cheng *et al.* 2021) and updated nanopore proteins (Karst *et al.* 2021). Meanwhile concurrent developments in library preparation and whole-genome amplification have enabled the use of decreased input DNA amounts (Schneider *et al.* 2021). A recent study found that the best lepidopteran genome assembly available at the time utilized a combination of HiFi data and Hi-C (Ellis *et al.* 2021).

Within genome assembly, accounting for genomic variation is largely a technical consideration. However, this variation is not uniformly or randomly distributed and is shaped by a range of evolutionary and demographic processes. One particularly challenging aspect of genome assembly is the resolution of highly divergent regions (HDRs; Kajitani *et al.* 2019), which often cannot be determined as allelic within the assembly process and requires supervised analysis (Roach *et al.* 2018). Genome assembly projects often aim to pre-emptively avoid this problem by severely inbreeding the source material to increase the proportion of genome homozygosity (The Heliconius Genome Consortium 2012; Nowell *et al.* 2017). However, the previous studies indicate that high levels of heterozygosity are often counter-intuitively maintained despite multiple generations of sib–sib inbreeding (The Heliconius Genome Consortium 2012; Nowell *et al.* 2017). A candidate for such an effect is the presence of overdominant or pseudo-overdominant loci. These loci, by various mechanisms, produce severe fitness consequences in a homozygous state. In the case of pseudo-overdominance, the presence of tightly linked recessive lethal mutations on different alleles prevents either haplotype from becoming homozygous (Charlesworth and Willis 2009). Alternatively, pseudo-overdominance may be produced by multiple linked mildly deleterious alleles, of which the cumulative effect is functionally equivalent to a single recessive lethal. Whatever the fundamental cause, these phenomena can also accumulate linked neutral variation, particularly in recombination cold spots (Zhao and Charlesworth 2016). These features appear to make pseudo-overdominance blocks a plausible candidate for the HDRs known to interfere with genome assembly (Waller 2021).

If HDRs can indicate regions experiencing particular forms of selection, failure to properly resolve them could impact downstream analyses, particularly the detection of balancing selection and overdominant loci. Since this bias is nonrandom, it may also affect comparative genome analyses, for example in instances of balancing selection predating speciation, or other forms of trans-species polymorphism, such as the well-studied MHC locus (Azevedo *et al.* 2015). Similarly, there may be common features of the genetic architecture that may be more likely to produce effects like overdominance or pseudo-overdominance at common ancestral regions. Nonetheless, the increasing quality of long-read sequencing, read lengths, and supporting data should help to mitigate the issue and enable evaluation of the scale of this problem across historic genome datasets. One method that is used widely in evaluating the completedness of genome assemblies is the use of highly conserved gene sequences that are consistently present as single-copy genes (Simão *et al.* 2015). However, in the context of HDRs and their putative sources, it is possible that these methods may be biased against representing genome regions that are more likely to harbor HDRs. One potential resolution to this is to find genome validation methods that do not rely on such cross-species inferences (Rhie *et al.* 2020).

Here, we investigate the complex trade-offs that are made in the choice of genome assembly algorithm using a long-read genomic dataset for the diamondback moth—*Plutella xylostella*, which was the subject of a previous major genome sequencing effort, culminating in the publication of an assembly in 2013 (GCA_000330985.1; You *et al.* 2013). The assembly strategy utilized the sequencing of fosmids, in order to mitigate the short-read lengths of Illumina sequencing. The authors report extensive structural variation based on alignments between their assembly and both the fosmids and a previously sequenced BAC (GenBank accession GU058050). The genome of *P. xylostella* therefore represents 2 distinct challenges to current long-read assembly methods, namely a large proportion of structural variation and a small amount of extractable DNA per individual. Our study includes the additional challenge of sequencing the heterogametic sex, containing the W-chromosome which has been shown to be highly repetitive and intractable to assembly (Traut *et al.* 2013).

## Methods

### Insect material origin and DNA extraction

Starting material was provided by Oxitec Ltd. (Abingdon, UK) from a lab colony that has been continuously cultured on artificial diet and is derived from the Vero Beach strain (Martins *et al.* 2012). Several lines were inbred in parallel by mating sib–sib pairs each generation. A 7 generation inbred family was selected for genome sequencing. DNA was extracted by phenol-chloroform from a pool of 15 sisters of the final inbred generation and a single male and female (Saccheri and Bruford 1993).

### Library construction and sequencing

The pooled DNA was sheared to 7 or 10 kbp. A subset was size selected at 15 kbp on the BluePippin (Sage Science, Inc.). In total, 66 SMRT cells were sequenced with P5-C3 chemistry on the RSII platform (Pacific Biosciences, Inc.). Reads were filtered according to subread length (>50 bp), polymerase read quality (>75 bp), and polymerase read length (>50 bp). Extracted DNA from the individual male and female was sheared and used for individual libraries followed by 2× 100-bp paired-end Illumina sequencing (Illumina, Inc.).

### Genome assembly parameters

We performed assembly using canu (version 2.1.1), flye (version 2.8.2-b1689), and wtdbg2 (version 2.5). These assemblies were subsequently polished with the same pacbio read-set for 2 iterations using quiver (version 2.3.3). As a preliminary step, we applied author recommended parameters for producing separated haplotypes in the presence of heterozygosity and subsequently used the resulting assembly with the highest rate of duplicated BUSCO genes (run as described below). For flye and wtdbg, we selected the default parameter result, for canu, we selected the assembly using the parameter set [genomeSize = 340m corOutCoverage = 200 correctedErrorRate = 0.040 “batOptions = -dg 3 -db 3 -dr 1 -ca 500 -cp 50”].

### Haplotype merging

We trialed 2 postassembly haplotype merging procedures, purge_dups and Haplomerger 2. Genomes processed with Haplomerger2 were first masked using windowmasker (version 20120730). A species-specific scoring matrix was inferred at 95% identity using the lastz_D_Wrapper.pl script included with Haplomerger2 (Huang *et al.* 2017). The masked genome and scoring matrix were then used to run scripts B1–B5 of the Haplomerger2 pipeline (version 20161205; Huang *et al.* 2017).

### Scaffolding

The preparation of HiC libraries was performed by Dovetail LLC using pools of starved larvae. HiC libraries were prepared as described by Kalhor *et al.* (2011). Both library preparations used the restriction enzyme DpnII for digestion after proximity ligation. Scaffolding and misassembly detection were performed by running the 3D-DNA pipeline on each of the haplotype merged assembly versions.

### Validation procedures

Here, we quantify the haplotype resolution processes using 2 independent methods. Firstly, we utilized the gene-based BUSCO score (version 5.0.0) with the “Lepidoptera odb10” database, consisting of 5,286 gene groups and augustus species model “heliconius_melpomene1.” Secondly, we utilized a combination of the stacked k-mer coverage histograms (a.k.a. spectra-cn) plots generated with KAT (version 2.4.2) and the read-based k-mer models produced by the genomescope R script. In brief, we used the R function “pmin” to intersect the assembly copy number coverage distributions with the modeled distributions from genomescope, specifically, the error distribution, and the heterozygous and homozygous components of the unique distribution. This provided a quantitative k-mer-based comparison of the haplotype resolution processes.

### Haplotype divergence assessment

We quantified the divergence between duplicated genes identified by the BUSCO analysis using an alignment-based and a supporting alignment-free method. The amino-acid sequences of duplicated BUSCO gene copies were aligned with MAFFT (version v6.864b), and subsequently translated into codon-based alignments with pal2nal.pl (version 14), followed by calculation of synonymous and nonsynonymous variants using the biopython function “cal_dn_ds.” For the alignment-free comparison, we used the full-genomic sequence (including introns) between the gene start and end coordinates identified by BUSCO and used the python package “alfpy” with a word size of 2 to calculate the Canberra distance (see 28).

## Results

### Insect materials, sequencing, and assembly

The material described in this study was inbred for 7 generations and is derived from a long-term laboratory culture, itself derived from the “Vero Beach” strain (United States). Fifteen sisters were pooled to meet minimum HMW DNA input requirements. Initial assemblies showed that substantial genetic variation was retained and was of sufficient complexity to produce multiple allelic sequence contigs from the same locus, inflating the total size way beyond the expected size of 338.7 (+/−1.1) Mbp, reported by Baxter *et al.* (2011). We subsequently trialed 2 approaches to resolve the redundant heterozygosity Haplomerger2 and purge_dups (Huang *et al.* 2017; Guan *et al.* 2020). After filtering 2,655,788 PacBio subreads remained (mean subread length = 7,301 bp, N50 = 10,398 bp, total bases 19.4 Gbp).

### Heterozygosity assessment

**Fig. 1.**
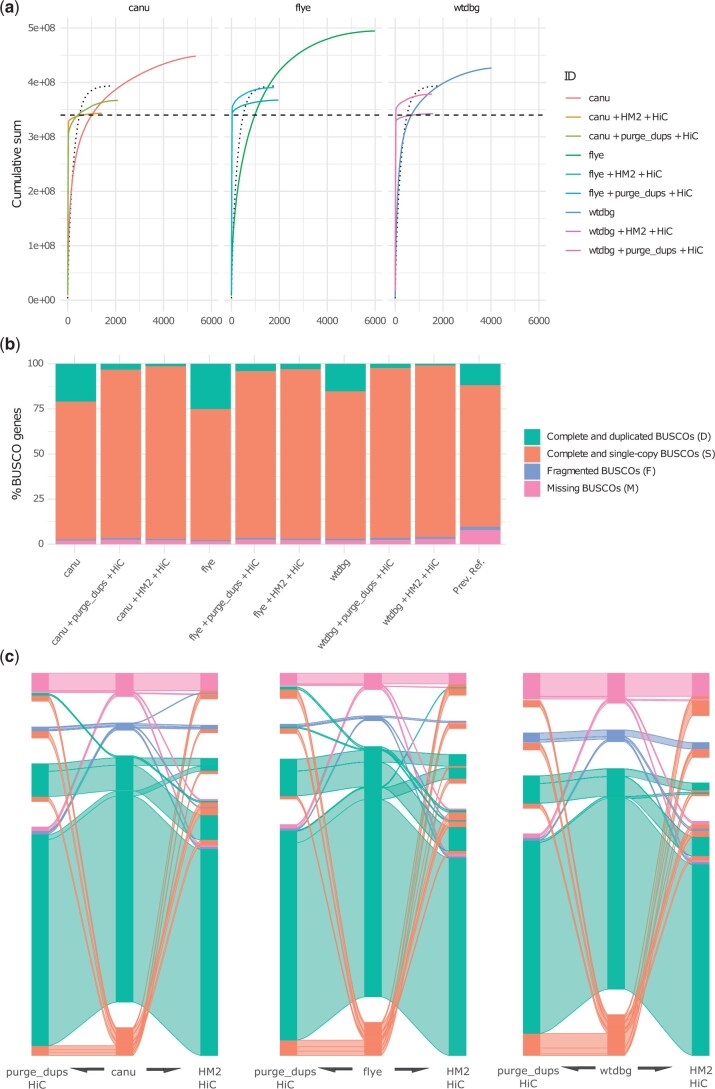
Contiguity and BUSCO content and of alternative genome assembly methods and the effects of removing putative allelic redundancy. In each panel, “canu,” “flye,” and “wtdbg” refer to the preliminary assemblies produced by each algorithm. “+ purge_dups + HiC” refers to these same assemblies with the additional application of the purge_dups program followed by HiC scaffolding or, Haplomerger2 followed by HiC scaffolding (a) depicts the differences in overall contig size and contiguity between the different methods. The dotted curve describes a previously published reference genome (accession: GCA_000330985.1). The dashed straight line indicates the estimated genome size from an independent flow cytometry estimate (Baxter *et al.* 2011). (b) Overall BUSCO scores from a database of 5,286 genes. BUSCO scores from the aforementioned accession are also included. (C) This image details the relationships of genes within these sets. Groups of genes are colored by BUSCO score in the initial assembly. BUSCO genes that are single copy and complete in all assemblies are omitted to emphasize differences between assemblies.

**Fig. 2.**
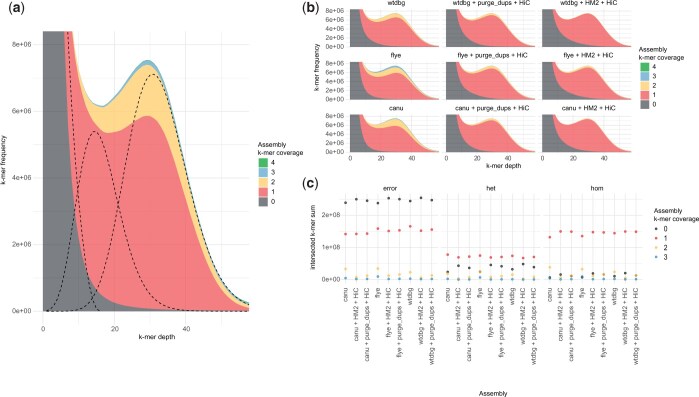
A k-mer-based validation of the alternative genome assembly methods and effects of removing putative allelic redundancy. a) An example of stacked k-mer distributions subdivided by assembly representation (spectra-cn plot) and an overlay of the modeled contributions of sequencing errors, heterozygous content and homozygous content (dotted lines from left to right, respectively). b) The spectra-cn plots for each of the assembly versions (c) shows the number of k-mers present in the intersections between the modeled k-mer content distributions and individual assembly coverage categories present in the spectra-cn plots.

All initial assembly strategies resulted in an over-inflated genome size, suggesting differing amounts of redundant haplotig sequence (Fig. 1a). We determined BUSCO results for the initial assemblies as evidence for the levels of allelic redundancy (measured as duplicated BUSCO genes) and overall completeness (Fig. 1, b and c). We also utilized k-mer-based methods. Firstly, a histogram of corrected PacBio reads, provided an initial estimation of genome heterozygosity as approximately 1.11%, which is moderately, but not exceedingly high for a North American sample [see You *et al.* (2020) for context] (Fig. 2a). Estimates from related individuals (nonpooled) were 0.54% for a related inbred male and 1.00% for a related inbred female (Supplementary Fig. 3). Stacked histograms colored by assembly coverage provided a qualitative assessment of genome assembly completeness and redundant allelic variation (Fig. 2b). Secondly, we intersected the modeled distributions of homozygous, heterozygous, and sequencing error content from genomescope with stacked histograms in order to make quantitative comparisons of the initial assemblies (methodology illustrated in Fig. 1a, data shown in Fig. 2c; Vurture *et al.* 2017).

The WTDBG2 assembly was the smallest in size (427 Mbp) and contig number (4,023) (Fig. 1a). It had the lowest number of duplicated BUSCO genes (805) and highest number of missing genes (110) (Fig. 1b). Consistent with the BUSCO results, WTDGB2 had the lowest number of homozygous k-mers duplicated in the assembly (Fig. 2b). But it also had the highest number of modeled error k-mers present (Fig. 2c). In contrast, the Flye assembly was the largest in size (494 Mbp) and contig number (5,985). It had the most duplicated BUSCO genes (1,324) and the least missing genes (88). Again the k-mer results show concordance with the highest number of homozygous k-mers present duplicated in the assembly. However, the number of modeled error k-mers present in the assembly was comparable with the canu assembly. Canu produced intermediate values in total size (448 Mbp) and contig number (5,341). Similarly, BUSCO results indicated an intermediate number of duplicated genes (1,105) and missing genes (106). k-mer results followed the same pattern except for error k-mers in the final assembly.

Both postassembly allelic redundancy approaches reduced the overall sizes of the assemblies and appear to follow the patterns observed in the initial assemblies, such that WTDBG2 still retains the lowest number of duplicated and highest number of missing genes in contrast with Flye. For each of the 3 starting assemblies, Haplomerger2 provided a greater reduction in total size and number of contigs compared to purge_dups (Fig. 1a. When applied to the canu assembly we also observe an increase in contiguity (Fig. 1a), due to a tiling effect produced when corresponding redundant heterozygous regions are merged at the ends of contigs (Supplementary Fig. 1).

Postassembly processing resolved most duplicated BUSCO genes to a single copy regardless of the initial assembly algorithm, however, in all cases, the number of missing BUSCO genes also increased (Fig. 1, b and c). We observe that purge_dups resolved some duplications that are not resolved by Haplomerger2 and vice versa (Fig. 1c). Similarly, genes that go from complete and single copy in the primary assembly to fragmented or missing after postprocessing are not necessarily the same across the 2 methods (Fig. 1c). This suggests that removal of redundancy is not simply, more or less “aggressive,” and that performance varies by algorithm depending on specific sequence properties.

### Comparison of heterozygosity assessments

Using the k-mer intersection approach described in Fig. 2, we produce a k-mer proxy of BUSCO genes for comparison. The proxy is calculated using the modeled homozygous k-mers (analogous to single-copy genes) and divides the occurrence of duplicated assembly k-mers by the sum of the single copy and duplicated assembly k-mers (Supplementary Table 2). Levels of percent duplication in the initial assemblies are remarkably concordant between the genic (BUSCO) and unbiased (k-mer) methods (Supplementary Table 2), with the exception of flye. This exception is likely due to the relatively higher occurrence of 3-copy redundancy observed with the flye assembly algorithm (Fig. 2b), which are not captured in our k-mer proxy measurement (BUSCO duplications do not distinguish 2 copy genes from >2 copy genes). However, after assembly postprocessing to remove redundant haplotigs, BUSCO genes appear to overestimate the efficiency of the procedures in comparison to the k-mer proxy. Across all methods and initial starting assemblies, the k-mer proxy shows consistently higher residual duplication than suggested by BUSCO genes (Supplementary Table 2).

### HiC misassembly detection and scaffolding

We observed the greatest overall number of detected misassembled region candidates in “canu + purge_dups” after 2 iterations of the HiC scaffolding pipeline 3D-DNA. The least misassembled region candidates detected after 2 iterations were in ‘wtdbg + HM2’, followed by ‘canu + HM2’ (Table 1). We find the greatest disparity in total misassembled region candidates between postprocessing methods in the canu assemblies. Furthermore, we find that “canu + purge_dups” produced the lowest final N50 value, whereas all other assemblies produced very similar results, though the metric is limited by karyotype at this resolution (Table 1).

**Table 1.** Number of misassembly region candidates detected within the 3D-DNA pipeline.

	Total size (initial size) Mbp	Pre-HiC N50 (Mbp)	Post-HiC N50 (Mbp)	Narrow misassemblies		Wide misassemblies		Total iteration 2 misassemblies	Total disaparity
				Iteration 1	Iteration 2	Iteration 1	Iteration 2		
canu + HM2	343 (448)	1.14	11.24	719	806	134	107	913	619
canu + purge_dups	367 (448)	0.62	9.49	794	1,247	164	285	1,532	
flye + HM2	368 (494)	0.41	11.42	1,065	1,225	185	115	1,340	55
flye + purge_dups	391 (494)	0.32	11.44	835	1,187	225	208	1,395	
wtdbg + HM2	343 (427)	1.83	11.1	673	721	127	92	813	382
wtdbg + purge_dups	378 (427)	0.94	11.49	827	993	204	202	1,195	

We used the default resolution parameters “wide_res = 25,000 bp” and “narrow_res = 1,000 bp.”

### Patterns of divergence between redundant alleles

**Fig. 3.**
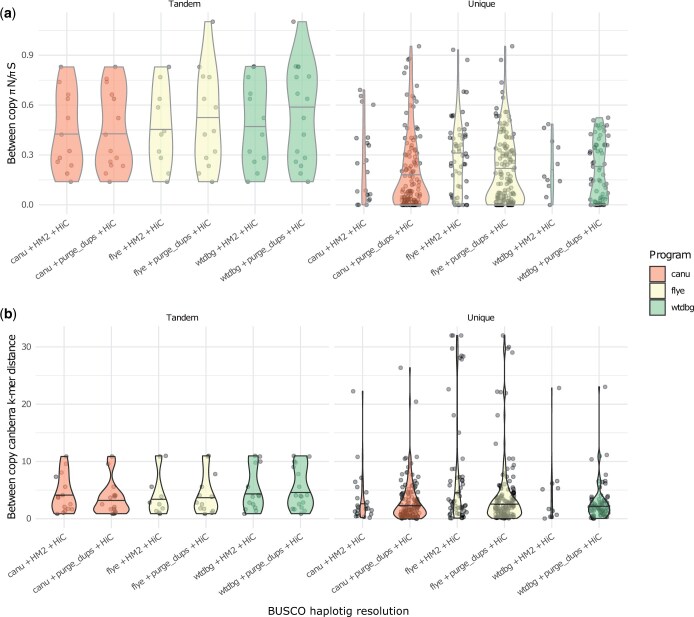
Quantifying divergence between duplicated BUSCO genes. a) shows the distribution of πN/πS scores for duplicated (N. copies = 2) BUSCO genes remaining after the application of purge_dups or Haplomerger2. b) This image shows an alignment-free quantification of the dissimilarity of intronic and exonic sequence between the same duplicated BUSCO genes (see *Methods* for details). Panels labeled “Tandem” indicate that the BUSCO copies were found on the same assembly contig, whereas “Unique” indicates that the copies were found on different assembly contigs.

For BUSCO genes that were duplicated in the initial assembly and subsequently reduced to single copy by the postprocessing methods, we broadly describe the variation between the copies using the ratio of nonsynonymous and synonymous nucleotide diversity and an alignment-free method using the entire genomic region (Zielezinski *et al.* 2019). We observed that the distributions of genes remaining after the application of purge_dups were more heavily weighted toward a low k-mer-based distance and a πN/πS ratio of 0 as compared to the distributions of genes deduplicated by Haplomerger2 (Fig. 3). We partitioned duplicated BUSCOs depending on whether they are present on the same initial assembly contig or not, as these genes may plausibly be real duplication events rather than haplotypic redundancy. The distribution of πN/πS between putative tandem duplicated BUSCOs (occurring on the same assembly contig) appears to be somewhat inflated in comparison to putative haplotig BUSCO genes. This pattern is also reflected in the overall genomic DNA k-mer distances (Fig. 3).

## Discussion

*Plutella xylostella* populations harbor large amounts of polymorphism (You *et al.* 2013, 2020). We observed a relatively low heterozygosity in our pooled data compared to other species; however, this individual should not be considered representative of the wild population due to severe inbreeding and prior laboratory domestication. Despite this apparently reduced heterozygosity, a large amount of redundant sequence remains after genome assembly, suggesting that the heterozygosity is largely colocalized in highly divergent alleles. This pattern may suggest regions of low recombination, enabling haplotypes to accumulate linked neutral variation and persist through drift. Alternatively, in the case of associative overdominance, neutral variation can accumulate alongside linked overdominant or pseudo-overdominant loci (linked deleterious recessives with opposing phase; Ohta and Kimura 1970). It is important that such regions are represented appropriately in genome assemblies, as downstream analyses involving mapping reads rely on both overall completeness and regions being present in a haploid state, although see (Armstrong *et al.* 2020) for how this is changing.

We tested 2 postassembly redundancy reduction procedures (Haplomerger2 and purge_dups) and found that Haplomerger2 generally appears to “resolve” more redundant sequence, at the expense of erroneous removal of nonredundant genome content and erroneous scaffolding of overlapping divergent regions. Both programs utilize a self-alignment step to detect haplotigs, purge_dups then implements a further QC step to these results by assessing the coverage of the identified haplotigs. For self-alignment, Haplomerger2 utilizes LASTZ and enables users to calculate and use a sample-specific scoring matrix, whilst purge_dups utilizes minimap2 with a fixed intraspecies scoring parameter (asm5). The parameterization reflects a balance in differentiating intralocus divergence, from interlocus paralog similarity. To give specific examples; ancient balancing selection vs relatively recent gene duplication or ancient balancing selection vs genetic convergence. The idealized genome assembly or redundancy removal pipeline can accurately differentiate these effects.

Genic analyses of assembly completeness such as BUSCO are widely used and relatively straightforward to apply, however, by definition they are limited to genomic regions containing coding sequences (Simão *et al.* 2015). The genes are highly conserved at the amino-acid level, suggesting that nonsynonymous substitutions are largely deleterious. Because of this, the surrounding genomic region (including noncoding variation) may be likely to harbor less variation than a neutral region, due to the action of background selection (Gilbert *et al.* 2020). In short, BUSCO genes are likely to inhabit (and help maintain) conserved genomic regions. The practical implication is that BUSCO genes, when utilized to assess the removal of redundant haplotigs, may systematically overestimate the effectiveness of the procedure, as they are unlikely to represent HDRs. Indeed, our results support the notion that before and after BUSCO duplication scores overestimate the removal of redundant haplotig sequences when compared to an analogous k-mer estimator. If BUSCO duplication results are liable to overestimate the haploid nature of a given draft genome assembly, it may hamper comparative genomic efforts to identify balancing selection or overdominance (which may have either common or independent origins).

Despite this potential limitation, BUSCO scores are still useful as a guide to assembly completeness. BUSCO scores also provided insights into assembly postprocessing, showing that, despite resolving more duplications than purge_dups, Haplomerger2 results do not completely overlap those of purge_dups. This indicates that both underlying methods are suboptimal and the results may be complementary. We also note that BUSCO results, particularly missing genes, are dependent on the optimization of input parameters. For example, the “–long” parameter can increase sensitivity at the cost of greater runtime. Similarly, the detection of BUSCO genes may differ between haplotypes, thereby underestimating the number of duplicated genes.

We demonstrate a supporting validation method, providing relative quantification of assembly accuracy, using overlaps between modeled k-mer distributions and the k-mer frequency histogram subdivided by numerical representation in the final assembly version. Whilst this assessment applies to any nonrepetitive genome region it only offers a general comparative measure between assemblies from the same read set and cannot determine appropriate representation at specific sites, due to stochasticity in read coverage. However, the ability to confidently determine truly heterozygous k-mers from homozygous will be increased in low-error, high-coverage read datasets, such as those currently being generated by projects like DToL (Darwin Tree of Life). This would offer an independent and unbiased validation method, but only with sufficiently high coverage to accurately partition the different k-mer peaks.

Our initial expectation was that a greater reduction in redundancy may correspond with an increase in detected misassembled region candidates, particularly in the case of Haplomerger2, which can join overlapping contigs (Supplementary Fig. 1). Instead, we find the opposite pattern, though this does not necessarily imply more accurate assembly representation, since complex regions may be absent from a final assembly altogether. For example, the lowest number of misassemblies (“wtdbg + HM2”), occurred alongside both the lowest putative allelic redundancy, but also the greatest values for missing BUSCO genes and het/hom modeled k-mers with 0× assembly coverage.

After an appraisal of the results of haplotig resolution, we compared the overall divergence of duplicated genes from the 2 methods. The results of purge_dups retained a greater proportion of low divergence haplotigs and this also corresponded to genes with a lower proportion of nonsynonymous substitutions. The remaining genes in both sets suggest that both methods did not resolve more greatly diverged sequences and that this divergence corresponded to elevated nonsynonymous substitutions relative to synonymous substitutions. Taken together with the high levels of coding sequence conservation intrinsic to BUSCO genes, this pattern would appear consistent with pseudo-overdominant regions generated by the linked arrangement of multiple deleterious nonsynonymous substitutions. However, additional investigations with supplementary data will be required to establish this with confidence and determine the processes responsible for these patterns.

### Conclusions

Highly divergent alleles can pose a challenge to accurate haploid reconstruction from noisy long-read data. Postprocessing can mitigate these problems somewhat to produce mosaic resolved sequences for reference purposes, however, results in our case are largely imperfect and present a set of complex trade-offs between assembly completeness, redundancy, and misassembly. Researchers producing or using genomes should be aware of these issues when using genome assembly data derived from noisy long reads, especially when investigating genomic regions likely to harbor significant linked variation. Our results lead us to the conclusion that unresolved HDRs may be widespread in draft genomes assembled from noisy long-read data and that BUSCO analyses may overestimate their resolution by postprocessing methods. Plausible causes include loci experiencing balancing-selection or overdominance effects that originated prior to speciation events, or that exist within a genetic architecture liable to parallel origins of these processes. This may impact comparative genomic studies that aim to identify or describe these evolutionary processes, however, further investigation is required to examine this.

Finally, as a recommendation to researchers utilizing similar data, we suggest that the optimal strategy is research question dependent. Our data show that there are complex trade-offs between gene set completeness, the presence and abundance of redundant haplotigs, and overall genome contiguity. We list some recommendations resulting from our dataset: (1) For comparative analyses of large-scale shared-synteny or chromosome-level structures, we would recommend wtdbg2 followed by Haplomerger2, however, it should be stressed that for this type of analysis researchers should supplement long-read data with HiC, both to extend contigs into larger-scale scaffolds, and also to correct any erroneous assembly, or postprocessing misjoins. When HiC data are available, the contiguity differences between different assemblers become less important, however, wtdbg2 followed by Hapolomerger2 should still reduce misleading interspecific alignment signals produced by residual haplotigs. (2) For comparative analysis of orthologs, the decision is complicated, as there is a trade-off between false-positive paralogs due to redundant haplotigs, vs false-negative missing genome content that is eliminated by redundancy removal procedures. (3) For analysis of a particular gene of interest, researchers can assemble their data with flye or canu, and process the resulting assembly with purge_dups. Reference to both the initial assembly and the postprocessed data should enable researchers to recover their gene of interest and examine whether any allelic variation is present. (4) For researchers wishing to produce a multipurpose reference assembly, with no specific research question, we would suggest producing detailed and transparent methods, such that subsequent users can understand the limitations and reanalyze for their specific purpose if necessary.

## Supplementary Material

jkac210_Supplemental_Material

jkac210_Supplemental_Figure_1

jkac210_Supplemental_Figure_3

## Data Availability

The read datasets generated during this study are available in the ENA database under accession PRJEB34571 (see Supplementary Table 1). All assembly versions are available at 10.5281/zenodo.5647466. Code provided at https://github.com/swomics/Plutella_genomes Supplemental material is available at *G3* online.
