# Supplementary material for: Recovering individual haplotypes and a contiguous genome assembly from pooled long-read sequencing of the diamondback moth (Lepidoptera: Plutellidae)
Source: G3 (Bethesda). 2022 Aug 18;12(10):jkac210. doi: 10.1093/g3journal/jkac210 (PMC9526047; doi:10.1093/g3journal/jkac210)
Supplement: jkac210_Supplemental_Material [file jkac210_supplemental_material.zip › jkac210_Supplemental_Material.docx]

# (Supplementary) Recovering individual haplotypes and a contiguous genome assembly from pooled long-read sequencing of the diamondback moth (Lepidoptera: Plutellidae)

Supplementary figure 1: A schematic of alignments produced by Haplomerger2, including two instances of contigs connected by a tiling effect, produced by merging corresponding heterozygous regions at the ends of contigs.

Supplementary figure 2: An overview of shared-synteny between Canu + Haplomerger2 + HiC and the silkworm (*Bombyx mori*), highlighting the high level of contiguity achieved. Alignments were generated with Cactus “--maxAnchorDistance 1000000 --minBlockSize 1000000” (Armstrong *et al.*, 2020)

Supplementary figure 3: K-mer distributions of independent Illumina sequencing libraries for a male individual (A) and female individual (B). Heterozygosity was estimated from the genomescope model as 0.54% and 1.00% respectively.

**Supplementary table 1:** Data accessions

| Data | Accession |
| --- | --- |
| PacBio reads | ENA ERR3569680 - ERR3569745 |
| Dovetail reads | ENA ERR3587405 - ERR3587406 |
| All assembly versions | Zenodo 10.5281/zenodo.5647466 |

**Supplementary table 2:** Comparison of BUSCO and k-mer methods for evaluating the removal of redundant haplotig sequences post-assembly.

|  | Complete single-copy BUSCO | Complete duplicated BUSCO | Single-copy homozygous k-mers | Duplicated homozygous k-mers | Percentage BUSCO duplication | Percentage k-mer duplication |
| --- | --- | --- | --- | --- | --- | --- |
| flye | 3844 | 1324 | 135,324,693 | 32,452,187 | 25.62 | 19.34 |
| flye + HM2 | 4972 | 155 | 147,586,644 | 11,722,640 | 3.02 | 7.36 |
| flye + purge_dups | 4889 | 212 | 146,709,666 | 16,058,833 | 4.16 | 9.87 |
| canu | 4038 | 1105 | 131,872,817 | 38,362,414 | 21.49 | 22.53 |
| canu + HM2 | 5062 | 70 | 149,832,199 | 6,281,000 | 1.36 | 4.02 |
| canu + purge _dups | 4938 | 170 | 149,190,205 | 12,463,520 | 3.33 | 7.71 |
| wtdbg2 | 4324 | 805 | 144,339,907 | 24,036,396 | 15.70 | 14.28 |
| wtdbg2 + HM2 | 5025 | 47 | 149,558,941 | 4,776,277 | 0.93 | 3.09 |
| wtdbg2 + purge_dups | 4980 | 119 | 148,899,603 | 12,943,479 | 2.33 | 8.00 |
