# Supplementary figures and images for: Recovering individual haplotypes and a contiguous genome assembly from pooled long-read sequencing of the diamondback moth (Lepidoptera: Plutellidae)
Source: G3 (Bethesda). 2022 Aug 18;12(10):jkac210. doi: 10.1093/g3journal/jkac210 (PMC9526047; doi:10.1093/g3journal/jkac210)

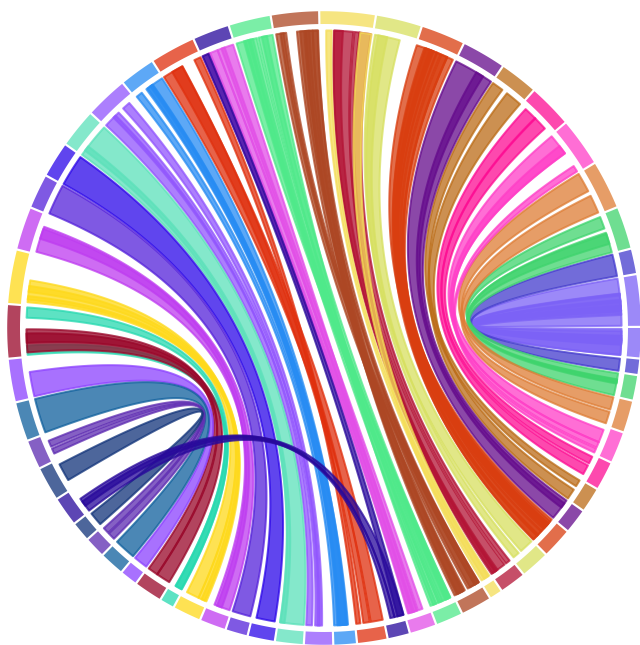

Supplement: jkac210_Supplemental_Material [file jkac210_supplemental_material.zip › jkac210_Supplemental_Figure_2.pdf]

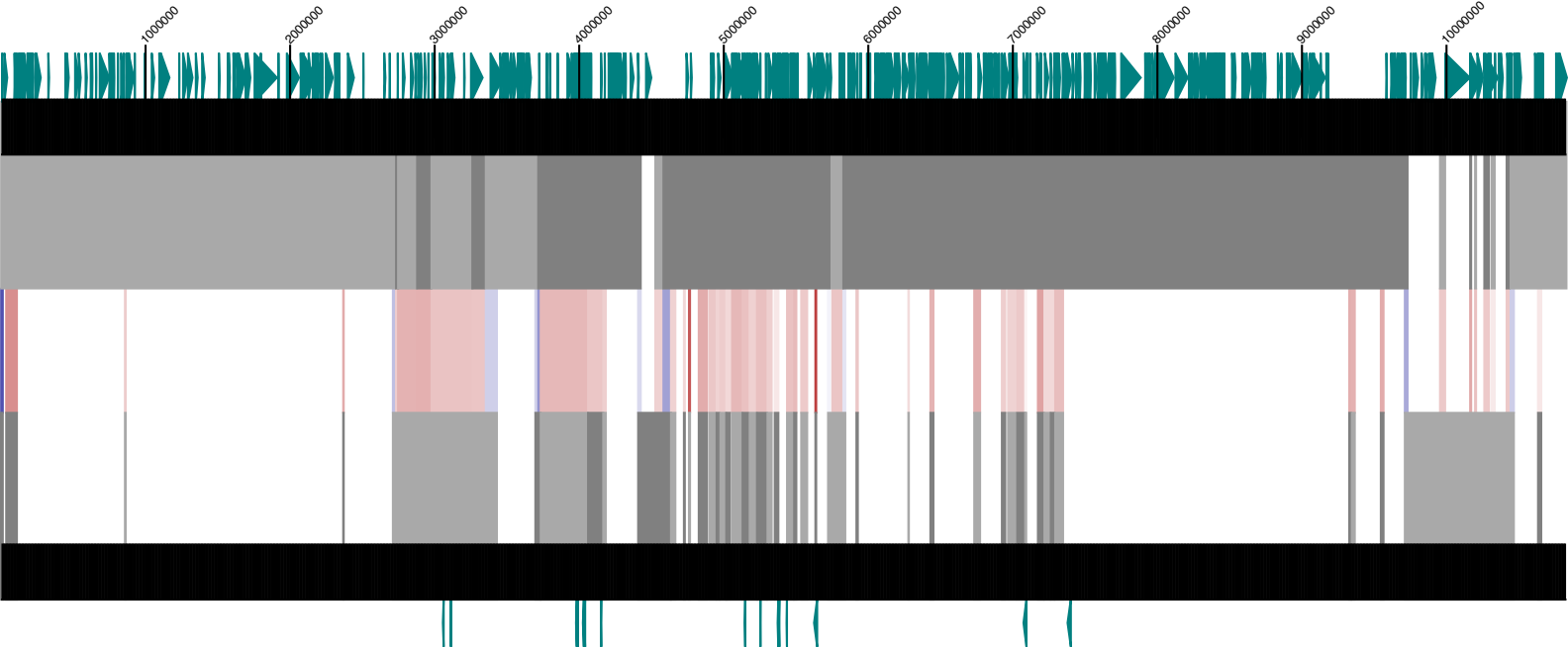

Supplement: jkac210_Supplemental_Figure_1 [file jkac210_supplemental_figure_1.pdf]

A

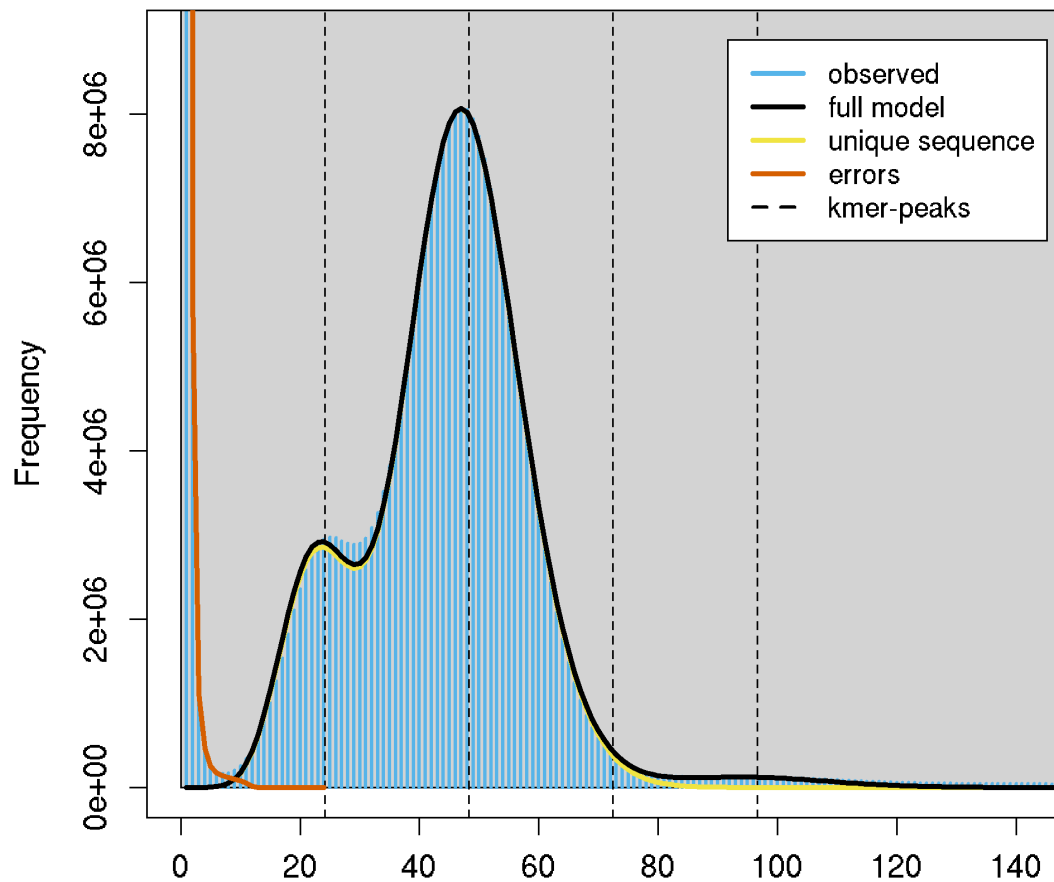

B

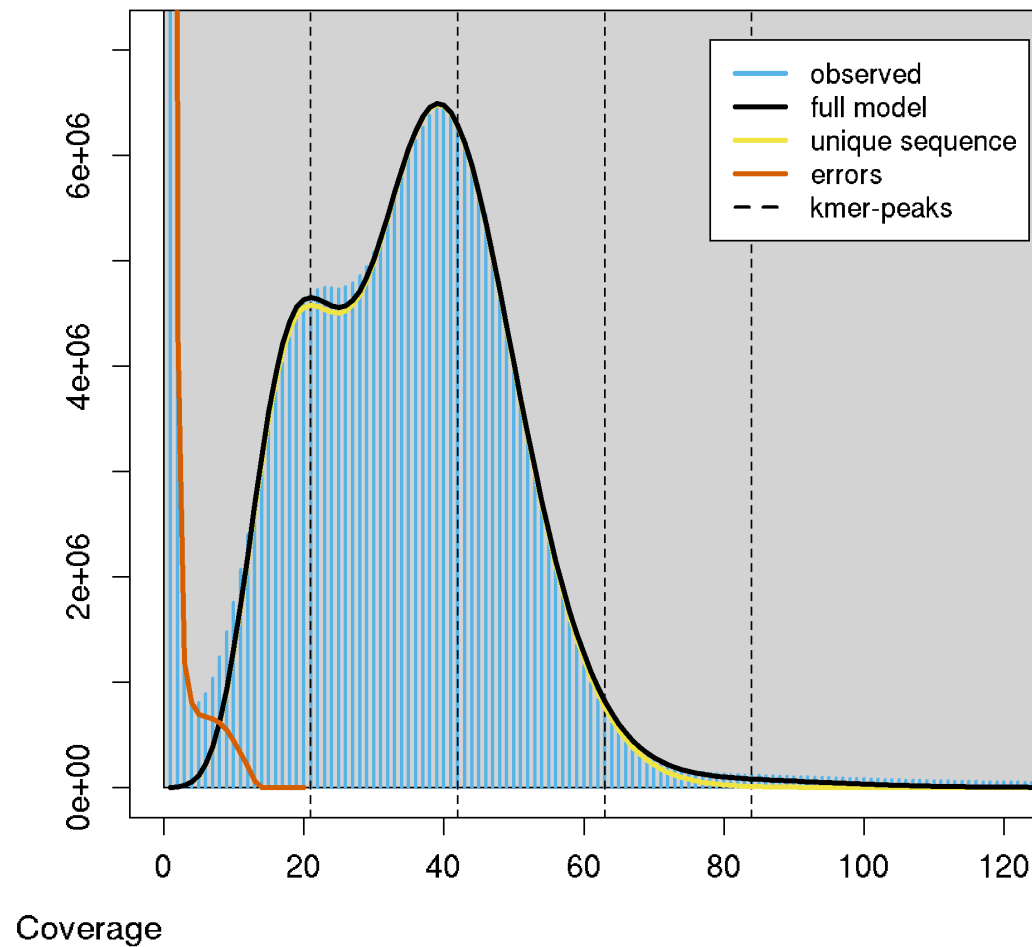

Supplement: jkac210_Supplemental_Figure_3 [file jkac210_supplemental_figure_3.pdf]
